# Study of morphology, chemical, and amino acid composition of red deer meat

**DOI:** 10.14202/vetworld.2017.623-629

**Published:** 2017-06-10

**Authors:** Eleonora Okuskhanova, Bahytkul Assenova, Maksim Rebezov, Kumarbek Amirkhanov, Zhanibek Yessimbekov, Farida Smolnikova, Almagul Nurgazezova, Gulnur Nurymkhan, Marilyne Stuart

**Affiliations:** 1 Department of Technology of Food and Light Industry Products, Shakarim State University of Semey, Semey City, Kazakhstan; 2 Department of Management of Technology Innovations and Veterinary Activity, Russian Academy of Staffing of Agro-industrial Complex, Moscow, Russia; 3 Department of Technologies of Production and Processing of Agricultural Products, Ural State Agrarian University, Yekaterinburg, Russia; 4 Canadian Nuclear Laboratories, Chalk River Laboratories, Chalk River, Ontario, Canada

**Keywords:** amino acid, composition, maral, red deer meat, quality, water-binding capacity

## Abstract

**Aim:**

The aim of this study was to evaluate red deer (maral) meat quality based on chemical composition, pH, water-binding capacity (WBC), and amino acid content.

**Materials and Methods:**

Maral meat surface morphology measurements were obtained by scanning electron microscopy. Active acidity (pH) was determined by potentiometry. Samples were analyzed for WBC by exudation of moisture to a filter paper by the application of pressure. Chemical composition (moisture, protein, fat, and ash fractions) was obtained by drying at 150°C and by extraction, using ethylic ether, and ashing at 500-600°C. The amino acid composition was obtained by liquid chromatography.

**Results:**

Maral meat, with a pH of 5.85 and an average moisture content of 76.82%, was found to be low in fat (2.26%). Its protein content was 18.71% while its ash content was 2.21%. The amino acid composition showed that lysine (9.85 g/100 g), threonine (5.38 g/100 g), and valine (5.84 g/100 g) predominated in maral meat, while phenylalanine (4.08 g/100 g), methionine (3.29 g/100 g), and tryptophan (0.94 g/100 g) were relatively low in maral meat compared to other meats. The average WBC was found to be 65.82% and WBC was found to inversely correlate with moisture content.

**Conclusion:**

Low-fat content, high mineral content, and balanced amino-acid composition qualify maral meat as a worthy dietary and functional food.

## Introduction

Nontraditional meat products are continually emerging on the market, notably products containing horse, deer, rabbit, ostrich, wild yak, and game meat [1-4]. Game meat tends to be preferred over the meat obtained from livestock animals [5]. The main reason for this is that game meat is considered a natural food. This is because wild animals are thought to feed as nature intended, and they are perceived as being strong (as they survived natural selection) and less stressed than livestock animals (as they live free and follow their instincts) [6,7].

**Figure-1:**
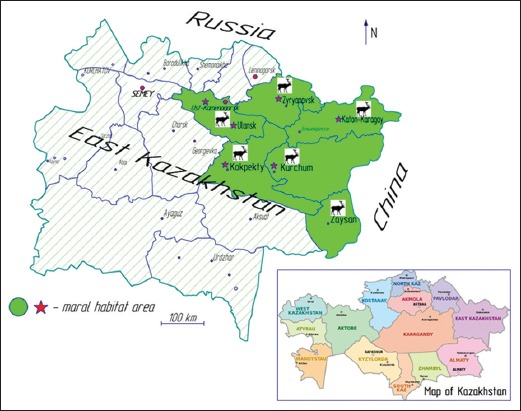
Maral habitat in East Kazakhstan.

Maral is one of the easternmost subspecies of red deer that is native to an area that covers part of Kazakhstan, China, Mongolia, and Russia [8]. Marals live in the Altai-Sayan mountains, southeast of Kazakhstan and in Predbaikal (Russia). The Republic of Kazakhstan marals are mainly inhabiting the east part of Kazakhstan (Figure-1) [9] and, as of 2012, the maral population is estimated at 3500 animals [10]. Maral meat is considered game meat.

In addition to the meat, that will be discussed later, blood and antlers are obtained from maral and spotted deer. This is because young antlers contain substances which have tonic effects, increasing the general state of health of humans. Antlers are also a source of protein, fat, and mineral elements, such as calcium (Ca), iron (Fe), silicium, phosphorus (P), sodium (Na), and potassium (K) [11]. The substance is also rich in amino acids such as glycine, proline, glutamic acid, lysine, leucine, and threonine [12]. Maral blood is of great pharmacological value. It can be used in the production of medicinal preparation, known as velvet antlers hematogen, for the treatment of nervous breakdown, metabolic disorder, catarrhs, gastritis, anemia, and other conditions [12]. Dried tails, leg tendons, penis, and 3-4 month embryos are also considered to have therapeutic value [13].

Maral meat is rich in minerals, essential amino acids, vitamins (5-10 times more than in beef), protein (it has a protein content of 18-20%) and it is low in fat (its fat content varies between 1.1 and 3.9% and the meat has a low cholesterol content) [14]. Maral meat also contains bioactive substances, ferments and hormones which are considered beneficial. The caloric value of maral meat is 944-1154 kcal per 100 g [15]. Kaimbayeva [16] determined that, while for the most part the mineral composition of maral meat is equivalent to beef, some elements are found in greater amounts in maral meet compared to beef. Maral meat is richer in Ca, fluorine, Fe, copper (Cu), zinc (Zn), and chromium compared to beef [17]. Maral meat is also a very valuable source of Vitamins: A, B, C, and E; as well as minerals: Fe, K, Ca, magnesium (Mg), Cu, Zn, and selenium [18,19]. The meat contains bioactive substances such as ferments and hormones, which can be beneficial to the weakened body. The meat represents 55-60% of the weight of the animal [20].

Due to its high Fe concentration, maral meat has a distinctive smell and sweetness associated with a metal taste. Serine, aspartic acid, glutamic acid, low molecular and volatile fatty acids also play a major role in the development of the meat flavor [21]. As we will see later, there are also some morphological differences between maral meat and the meat of livestock animals.

The purpose of this study is to evaluate red deer (maral) meat quality based on chemical composition, pH, water-binding capacity (WBC), and amino acid content.

## Materials and Methods

### Sampling

A total of 5 kg (2.5 kg from Ulan and 2.5 kg from Ust-Kamenogorsk city) of meat was collected for analysis. 10 samples of maral meat (*Musculus longissimus dorsi*) were obtained from the two local trade markets located in the Ulan settlement, and 20 samples from the three markets located in Ust-Kamenogorsk city. The markets are within the East Kazakhstan region (Figure-1). The meat was transported to the laboratory where it was stored at 2-3°C. The next day, the meat samples were grinded, mixed, and immediately used for analysis.

### Surface morphology and pH measurements

The surface morphology of the maral meat was studied using a scanning electron microscopy technique. The instrument used was a low-vacuum raster electron microscope “JSM-6390 LV” (JEOL, Japan). Active acidity (pH) was determined using a potentiometer method. A pH-tester 340 (Infraspak-Analit, Russia) was used to obtain the information. This was done simply by dipping the two electrodes into a solution and taking a reading. The solution was prepared as follows: The meat samples was minced and mixed with (distilled-deionized water in the ratio 1 part of meat: 10 parts of water. The pH reading was obtained after 30 min of infusion at 20°C.

### WBC

The method used to determine the WBC of the samples is based on exudation of moisture to a filter paper by the application of pressure. The moisture absorbed by the filter paper is evaluated based on the spot area on the filter paper. Specifically, for each sample, 0.3 g of minced meat was placed on a 15-20 mm diameter disk plate on a Mettler Toledo electronic balance, (Mettler Toledo, Switzerland). The meat was then transferred onto an ash-free filter (Munktell Filter AB, Sweden) and placed on a glass or plexiglass plate. The sample was covered with the same kind of filter before a 1 kg load was carefully placed on top of the meat. The weight was left for 10 min. Once removed, the top filter was pulled of and bound water was calculated, as described below (Equations a and b). The filter was scanned using an Xpress M2070 scanner (SAMSUNG, Japan) after the contour of the wet spot was traced on the filter. The area was calculated using the “Compas-3D V-10” software [22].

*X_1_=(A-8,4B)·100/m_0’_* (a)

*X_2_=(A-8,4B)·100/A;*(b)

Where *X_1_ –* bound water content, expressed as % of meat;

*X_2_ –* bound water content, expressed as % to total water;

*B* – wet spot area, cm^2^;

*m_0_ –* sample weight, mg;

*A –* total content of moisture in the sample, mg.

### Chemical composition

The determination of the chemical composition of meat was based on the determination of the following constituents: Moisture, fat, ash, and protein. To determine water content, a 2-3 g aliquot of each sample of meat was weighted to the nearest 0.001 g using a Mettler Toledo electronic balance (Mettler Toledo, Switzerland) and placed into a metallic cup (IngoLab, Russia). It was then dried for 1 h, in a drying oven SNOL 67/350 (Umega, Latvia), at a temperature of 150°C. The moisture content was calculated using Equation c, according to the standards GOST 9793-74 [23] and GOST R 51479-99 [24].

*x*_1_=(*m*_1_−*m*_2_)·100/(*m*_1_−*m*) (c)

Where, *x_1_* – moisture content, %;

*m_1_* – weight of sample with cup before drying, g;

*m_2_* – weight of sample with cup after drying, g;

*m* – weight of cup, g.

After moisture determination, each dried sample was moved to a glass cup. Then, 15 ml of ethylic ether (Chemically pure 100%, Skat, Kazakhstan) was poured into the glass cup and the contents were mixed for 3-4 min. During the extraction process, the ethylic ether containing the fat residues was poured out and replaced with fresh ethylic ether. After 4-5 repetitions, the ethylic ether was evaporated at room temperature. The cup containing the fat depleted sample was dried at 105°C for 10 min. According to the standard GOST 23042-86 [25], the fat content was calculated using Equation d.

*x*_2_=(*m*_1_−*m*_2_)·100/*m*_0 _(d)

Where, *x_2_* – fat content, %;

*m_1_* – weight of cup and dry sample before extraction, g;

*m_2_* – weight of cup and sample after extraction, g;

*m_0_* – weight of cup, g.

To obtain the ash content, the sample from which the fat was extracted was placed into a weighted and preheated (to 150°C) crucible (50 cm^3^, Mankor, Ukraine). Then, 1 ml of Mg acetate (Purity 98%, Labofarma, Kazakhstan) was added to the crucible, and the mixture was burned on an electric hot plate. After that, it was placed into a muffle furnace set at a temperature of 500°C-600°C (SNOL 7.2/1100, Umega, Lithuania) for 30 min. The ash content was calculated following Equation e:

*x*_3_=(*m*_1_−*m*_2_)·100/*m*_0 _(e)

Where, *x_3_* – ash content, %

*m_1_* – weight of ash, g;

*m_2_* – weight of Mg oxide, obtained after the mineralization of Mg acetate, g;

*m_0_* – weight of sample, g.

The protein content was assayed according to the standard GOST 25011-81 [26] and calculated using Equation f.

*x*=100−(*x*_1_+*x*_2_+*x*_3_) (f)

Where, *x* – protein content, %

*x_1_* - moisture content, %;

*x_2_* – fat content, %;

*x_3_* – ash content, %.

Liquid chromatography was used to quantify amino acids. The instrument used was a “Shimadzu LC-20 Prominence” liquid chromatography system (Shimadzu, Japan) equipped with fluorometric and spectrophotometric detectors. The chromatographic column used was SUPELCO C18, 5 µm (Sigma-Aldrich, USA) offering a surface area of 200 m^2^/g. The chromatographic analysis was performed under a linear gradient with an eluent flow rate of 1.2 ml/min, and the column was heated in an oven at 400°C. Amino acids were detected using fluorometric and spectrophotometric detectors at wavelengths of 246 nm and 260 nm following acidic hydrolysis and treatment with a phenylisothiocyanate solution in isopropyl alcohol to give phenylthiohydantoins.

### Statistical analysis

Differences between samples were evaluated using the t-test. The differences were considered to be statistically significant at p≤0.05. The statistical analysis was performed using the free software R 3.02 (R Core Team, 2013).

## Results and Discussion

**Figure-2:**
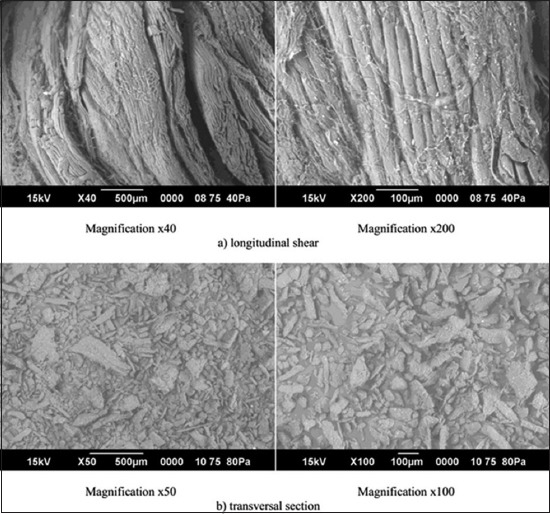
Images of longitudinal and transversal sections of maral muscle tissue obtained by scanning electron microscopy.

Images of the morphological microstructure of maral meat are presented in Figure-2. The maral muscle tissue longitudinal shear images show the length of the straight and curved muscle fibers. Images of the transversal sections show that each fiber has a polygonal structure. Looking at the muscle fiber size, it was noted that maral meat possesses a juiciness that is not seen in beef. In fact, the larger amount of moisture in maral meat compared to beef explains this difference [16]. Table-1 presents the moisture data obtained for maral meat during this study and, for comparison, shows values that have been reported for beef and horse meat. Maral meat was found to contain significantly more moisture compared to the other two meat types. One of the important meat quality indicators is its moisture content. Juiciness, tenderness, losses during heat treatment (or cooking), and appearance are all dependent on the ability of meat to hold or bind water. As shown in Table-2, the average WBC for maral meat was 65.82% which was found to be lower than previously reported beef (69.02%) [27] and horse meat (67.88%) values [28]. As for the moisture content, the differences were statistically significant. The WBC values were inversely correlated to moisture content. Proteins in muscle tissue are likely modulating the WBC [29,30].

**Table-1:** Chemical composition of beef, horse and maral meat, %.

Indicator	n	Beef [27]	Horse meat [28]	Maral meat
Moisture	10	73.81±0.98*	69.64±0.75*	76.82±1.16
Protein	10	13.70±0.25*	19.58±0.34*	18.71±0.27
Fat	10	10.29±0.17*	9.91±0.14*	2.26±0.03
Ash	10	1.15±0.02*	1.00±0.01*	2.21±0.04
Energy value (kcal)	10	147.4	167.1	91.04

* Indicate that the values are statistically different from the maral meat (t-test, p<0.05). Results are expressed as mean±SD. n=number of samples, SD=Standard Deviation

**Table-2:** pH and water-binding capacity of maral meat.

Value	n	Beef [27]	Horse meat [28]	Maral meat
PH	10	5.73±0.07	5.84±0.06	5.85±0.11
WBC, %	10	69.02±1.28*	67.88±0.82*	65.82±0.77

* Indicate that the values are statistically different from the maral meat (t-test, p<0.05). Results are expressed as mean±SD. n=number of samples, SD=Standard Deviation, WBC=Water-Binding Capacity, WBC=Water-Binding Capacity

**Figure-3:**
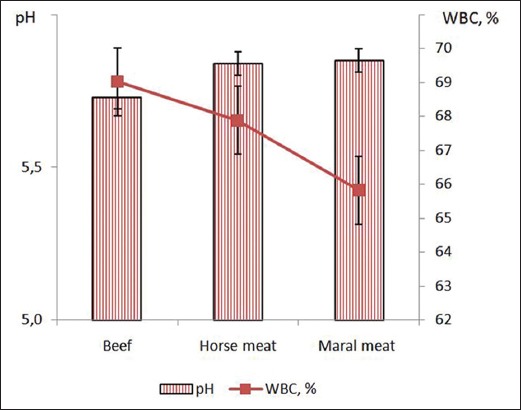
pH and water-binding capacity of beef, horse meat and maral meat.

In broad terms, the nutritional value of meat, however, depends on the quantitative ratios of moisture, protein, fat, and minerals. More specifically, it is also a function of the contents in essential amino acids, polyunsaturated fats, B vitamins as well as macro- and microelements. Tables-1 and 2 summarize differences in chemical composition in terms of protein, fat, and ash contents between maral meat, beef and horse meat to allow for a comparison of their respective nutritional value. Table-1 presents information regarding the protein, fat, and ash contents and Table-2 presents the pH data (Figure-3).

In comparison to the other meat types, maral meat was found to be low in fat. On average, maral meat’s fat content was found to be 2.26% while 9.91% was obtained for horse meat [28] and 10.29% was obtained for beef [27]. Maral meat contains much less fat than beef and horse meat (by factors of 3.0 and 2.8, respectively). Maral meat is very lean because the lipids are mainly deposited in the subcutaneous fat layer of the animal while, in the livestock animals, fat deposits are not only in the subcutaneous fat layer but also in the muscular fraction [31].

The protein content of maral meat, at 18.71%, was found to be higher than beef (13.70%) and lower than horse meat (19.58%) [27,28].

A higher content of ash was obtained for maral meat (average of 2.21%) compared to beef (1.15%) [27] and horse meat (1.00%) [28]. This demonstrates abundance of minerals. The results obtained in a previous study [14] showed that maral meat is a very suitable source of micronutrients such as: K - 3045.30mg/kg, P - 592.12mg/kg, Mg - 224.07mg/kg, Na - 217.94mg/kg, Ca - 77.28mg/kg, Fe - 38.39mg/kg, aluminium - 36.58 mg/kg, Zn - 30.04 mg/kg, manganese - 6.92mg/kg, Cu - 1.40mg/kg, and nickel - 0.30mg/kg. The average maral meat pH value was 5.85. This value was similar to values previously reported for horse meat and beef.

The chemical composition results, obtained in this study, were compared with other data reported in different sources (Table-3). According to the deer meat composition reported in other studies, the protein, fat and ash content ranged from 17.4% to 22.4%, 0.3% to 12.13% and 0.5 to 4.54%, respectively. The results of this study, with average values of 18.71%, 2.26% and 2.21%, respectively, compared very well. It stands to mention the work of Jussupbekova, published in 2007, [32] for which maral meat was sampled from East Kazakhstan in 2003-2006. This work is of particular interest as the maral samples were obtained from the same region as the samples obtained to carry out the current study. Jussupbekova showed a slightly higher protein content (19.4%) and lower fat (1.4%) and ash (0.7%) contents, compared to the current study results. The differences in composition are likely due to factors such as differences in climatic conditions, landscape, living environment, feeding conditions, type of deer or meat cuts.

**Table-3:** Chemical composition of deer meat previously reported in the literature.

Source	Moisture	Protein	Fat	Ash
This study	76.82	18.71	2.26	2.21
Kaimbayeva, 2014 [16]	78.2	17.4	3.2	1.2
Ossipova, 2013 [31]	74.9	21.6	2.5	1.0
Ketselashvili *et al.*, 2011 [5]	76.4	20.9	2.4	0.5
Strazdina *et al*., 2013 [33]	70.6	22.4	1.9	1.1
Malofeev *et al*., 2012 [34]	76.7	21.4	0.3	1.6
Okhremenko and Lee, 2005 [35]	78.0	20.0	1.1	0.9
Daszkiewicz *et al*., 2009 [19]	-	22.01	0.56	1.1
Dahlan and N. Hanoon, 2008 [36]	70.62	21.86	12.13	4.54
Lisitsyn *et. al*., 2011 [37]	70.3	21.6	6.4	1.0
Jusupbekova, 2007 [38]	78.5	19.4	1.4	0.7

As the total protein content does not fully quantifies nutritional value, it is necessary to determine the amino acid composition. Amino acids are heterofunctional compounds which form the structure of proteins. They are organic compounds containing amine (-NH_2_) and carboxyl (-COOH) functional groups, along with a side chain (referred to as R group) which is specific to each amino acid. Thus, the balance and full-value of the amino acids in the proteins determine the physiological effect to the human body [38].

The amino acid content of maral meat, beef and horse meat is presented in Table-4. Although the content of some amino acids differ markedly depending on the meat cuts and the amount of associated connective tissues, in the current study, maral meat was found to contain more lysine, threonine and valine compared to the beef and horse meet. In maral meat, the isoleucine content is higher than in beef but is lower than in horse meat. Phenylalanine, methionine, and tryptophan represent a lesser proportion of the amino acids found in maral meat compared to beef and horse meat. Except for methionine and tryptophan, maral meat’s composition in amino acid compares favorably to the food and agriculture organization (FAO) scale.

**Table-4:** Essential amino acids content in meat of slaughtered animal, g/100 g of product.

Essential amino acids	FAO scale		Beef [27]		Horse meat [28]		Maral meat	
			
	g/100 g	AS, %	g/100 g	AS, %	g/100 g	AS, %	g/100 g	AS, %
Valine	5.0	100	5.0	100	5.5	110	5.84	119
Isoleucin	4.0	100	4.8	120	6.7	154	5.83	146
Leucine	7.0	100	8.1	116	8.3	119	7.40	106
Lysine	5.5	100	8.9	162	8.2	150	9.85	179
Methionine	3.5	100	3.5	100	3.7	106	3.29	94
Threonine	4.0	100	4.6	115	4.7	118	5.38	135
Tryptophan	1.0	100	1.1	110	1.2	120	0.94	94
Phenylalanine	6.0	100	4.5	75	5.5	92	4.08	68
Total	36.0		40.5		43.8		42.61	

AS=Amino acid score

Lysine, in which maral meat is rich in, is needed for normal bone shaping and children growth. It helps to better absorb Ca and contributes to the maintenance of normal nitrogen cycling in humans. Lysine is also involved in synthesis of antibodies, hormones, and ferments. It contributes to collagen formation and tissue recovery [39].

It is known that threonine, another amino acid that is found in high levels in maral meat, improves the cardiovascular and immune systems as well as liver condition. Furthermore, threonine is involved in glycine and serine synthesis. This amino acid strengthen the connective tissues and muscles, including the heart muscle [38].

Tryptophan, which is found in greater amounts in beef and horse meat compared to maral meat, is a factor of vitamin PP synthesis. A deficiency in this amino acid can cause pellagra. A tryptophan imbalance can lead to serious illnesses such as tuberculosis, cancer, and diabetes [40]. Tryptophan is an essential amino acid that is usually present in lean tissue and in lesser amounts in connective tissue and muscles, which are rich in oxyproline [41].

Methionine, also found in greater quantities in beef and horse meat compared to maral meat, is one of the main building blocks in the human body and it is essential to prevent vitamin B12 deficiency [42].

One important factor to consider during the formulations of food products is the biological value of the proteins that depends on the balance of amino acid. As the human body is only able to produce nine (histidine and the 8 listed in Table-4) of the 22 amino acids, a deficiency in even one of the essential amino acids will lead to the failure of the protein synthesis and other biological substances [43]. Thus, the daily nutrition should contain enough of these amino acids [39,44]. According to the data presented in Table-4, the maral meat contains 42.61 g of amino-acids per 100 g of meat which is higher than beef but less than horse meat.

## Conclusion

Maral meat was found to have a low fat content, high mineral content, and balanced amino-acid composition. This qualifies maral meat as a good dietary and functional food. Because of the wide use of beef in the production of meat products, the quantity of fat consumed is relatively high. When meat represents a large fraction of the diet, leaner alternative to beef, like maral meat, are desirable. Using maral meat in the formulations of meat products could therefore contribute to decreasing fat intake while increasing nutritive and biological value.

## Authors’ Contributions

This work came from active collaboration between researchers: EO, BA, MR, designed the study, developed the methodology, performed the analysis, and wrote the manuscript. ZY conducted the scanning electron microscope analyses and assisted with data analysis. FS, AN, GN performed the chemical and amino acid composition determinations. MS, KA provided helpful feedback on an early draft of the paper. MS proofread and corrected the English. All authors read and approved the final manuscript.
